# Detection of SARS-CoV-2 antibodies in pediatric kidney transplant patients

**DOI:** 10.1186/s12882-021-02325-x

**Published:** 2021-04-07

**Authors:** Alanoud Alshami, Rabab Al Attas, Ahmad Azzam, Amani Mohammed, Norah Al-Quhaidan

**Affiliations:** 1 Division of Pediatric Nephrology and Kidney Transplant, Multiorgan Transplant Center, King Fahad Specialist Hospital-Dammam/Saudi Arabia, Dammam, Saudi Arabia; 2 Division of Immunology, Department of Pathology and Laboratory Medicine, King Fahad Specialist Hospital, Dammam, Saudi Arabia, Dammam, Saudi Arabia

## Abstract

**Background:**

The seroprevalence of SARS-CoV-2 infection has been studied in immunocompetent children. However, data in the pediatric kidney transplant population (PKT) are lacking.

**Methods:**

Using two commercial immunoassays that measured IgG antibodies against SARS-CoV-2 spike protein and IgG against the nucleocapsid (N) protein, we screened 72 PKT recipients who attended the outpatient clinic for routine blood work. The majority of patients with positive serology underwent an additional serology test at least once during subsequent clinical follow-up. Patients were confirmed to have SARS-CoV-2 infection if they had two positive tests.

**Results:**

Eight patients out of the 72 screened (11.1%) had positive results for SARS-CoV-2 IgG antibodies in both serological tests. Of those who tested positive, 4 had positive SARS-CoV-2 PCR results before screening. All patients were asymptomatic or had a history of mild symptoms. All tested patients had persistently positive antibodies at a median follow-up time of 75 days (IQR, 44.5, 86.5 days). One patient had a positive PCR test at 75 days and a positive serology test at 120 days post infection.

**Conclusion:**

The seroprevalence of SARS-CoV-2 was relatively high (11.1%) in our population. Although all patients were asymptomatic or mildly symptomatic, they mounted a strong humoral immune response that persisted for a few months despite being on triple immunosuppressants. These findings have positive implications regarding vaccination efficacy in this group.

## Introduction

The coronavirus disease 2019 (COVID-19) pandemic is considered a public health emergency. Despite all efforts and policies to contain it, severe acute respiratory syndrome (SARS-CoV-2) infection continued to spread rapidly. As of October 8, 2020, COVID-19 has led to over 1 million deaths and 3.5 million cases worldwide [1] Approximately 85% of cases will be mild, while 10% will have moderate disease requiring hospitalization, and 5% will have severe disease requiring ICU admission [2]

As pediatric solid organ transplant recipients are considered a high-risk group and are prone to more severe viral, bacterial, and fungal infections, this pandemic has raised concerns among the transplant community about managing this vulnerable group. In the early days of the pandemic, COVID-19 was thought to be more severe among immunocompromised patients. The fear of more severe disease has limited kidney transplant surgeries to those that are urgent and has led to the closure of many living transplant programs [3], including ours. This perceived risk in transplant patients is an extrapolation of data from other viruses. Unlike influenza and adenovirus [4], SARS-CoV-2 does not seem to preferentially affect immunocompromised patients. There is emerging evidence that immunosuppressed children exhibit mild disease [5–7]. This mild disease course could be explained by the role of immunosuppressants in damping of the dysfunctional or hyperimmune response that occurs in the later stages of the infection, leading to lung tissue injury and acute respiratory distress syndrome (ARDS) [8]. Although we are one year into the pandemic, little is known about the effect of COVID-19 on the pediatric kidney transplant (PKT) population.

To better understand the SARS-CoV-2 infection prevalence, clinical presentation and the rate of seroconversion in the PKT population, we aimed to perform universal SARS-CoV-2 serological testing in all our pediatric kidney transplant patients attending outpatient clinics.

## Methods

### Study design and subject enrollment

This study was approved by the institutional review board of King Fahad Specialist Hospital -Dammam/Saudi Arabia (IRB Number: PED0134). Verbal consent was obtained from all parents, and additional assent was obtained from those who were more than seven years old.

This study was a single-center cross-sectional study aiming to study the seroprevalence of SARS-CoV-2 in a pediatric cohort of kidney transplant recipients. All PKT recipients ≤17 years of age who attended an outpatient clinic visit or came to the hospital laboratory at King Fahad Specialist Hospital-Dammam for routine post-transplant blood work between August 15th and October 12th, 2020 were enrolled. Data on demographics, immunosuppressant use, primary disease, time since transplant and SARS-COV-2 PCR results were collected from electronic records. Two extra milliliters of blood for serology testing was drawn from all patients during routine blood work.

All children enrolled were retrospectively screened for COVID-19 symptoms in the last 3 months before enrollment through the clinic triage system or phone call surveys.

The symptom checklist included fever> 38 °C, cough, fatigue, shortness of breath, sore throat, runny nose, headache, abdominal pain, diarrhea, anosmia, and ageusia.

Serology tests were prospectively repeated for follow-up titers if the patient had initial positive serology, and a follow-up clinic visit or follow-up laboratory work were conducted for those with positive PCR who did not show evidence of seroconversion in the initial sample. Parents and/or patients were informed about the positive SARS-CoV-2 serology results and were asked again about any history of COVID-19 symptoms in the past 3 months or history of close contact with confirmed COVID-19 cases. They were also asked to perform PCR tests for their children if one of the serology tests was positive.

As the SARS-CoV-2 seroprevalence in the pediatric transplant population is not well determined and expected to be low, the positive predictive value of a single serological assay might not be accurate. To improve the diagnostic accuracy of the serological test, we used two different FDA-approved commercially available serological assays that test different SARS-CoV-2 protein components. Assays with dual target antigens have been shown to have better predictive abilities [9]. Only patients with positive results for both tests were deemed to have SARS-CoV-2 antibodies.

### Serological essay for SARS-COV-2 antibodies

#### Diasorine Liasion® SARS-COV-2 S1/S2 IgG assay

This assay is a quantitative chemiluminescent immunoassay (CLIA) against SARS-CoV-2 spike proteins S1 and S2 with a reported sensitivity of 97.6% at > 15 days, a specificity of 99.3% and a positive predicted value (PPV) of 87.5% [10]. The test was performed as per the manufacturer’s instructions. Readings > 15 AU/ml were considered positive, and readings < 12 AU/ml were considered negative; the upper detectable limit was 400 Au/ml as per the manufacturer’s manual.

#### Abbott architect SARS-CoV-2 IgG

This assay is a qualitative chemiluminescent microparticle immunoassay (CMIA) intended to measure IgG antibodies against SARS-COV-2 nucleocapsid (N) protein. As per the last EUA-authorized serology test performance, the Abbot test has 100% sensitivity, 99.6% specificity, and 93.4% PPV [10]. All assays were performed according to the manufacturer’s protocols**.** Samples were interpreted as positive or negative according to the manufacturer’s instructions [11].

## Results

### Demographics and clinical characteristics

The demographics and clinical characteristics of the entire cohort are shown in (Table 1). Of the 72 PKT patients screened by serological testing for SARS-CoV-2, 12.5% (9 of 72) were found to have positive SARS-CoV-2 IgG in at least one test. A total of 88.9% (8 of 9) patients had positive results on both tests. Notably, 4 patients out of the tested patients had a history of a positive PCR before serological testing. Two out of the four (50%) patients who had positive PCR were completely asymptomatic, and a test was performed for elective admissions, one for kidney biopsy for new onset de novo donor-specific antibodies (case 1) (Table 2) and the other for investigations for rising creatinine.

**Table 1:** Demographics and Clinical Characteristics of the 72 transplant patients enrolled

Characteristics	N (%)
**Sex**	
Male	44 (61.1%)
Female	28 (38.9%)
**Age** (*μ* ± *SD*)	9.83 ±3.87
**Primary disease**	
Congenital Nephrotic Syndrome	22 (30.6%)
Hypoplastic/Dysplastic kidney disease	19 (26.3%)
Obstructive Uropathy	10 (13.9%)
Nephronophthisis	5 (6.9%)
Idiopathic FSGS	4 (5.6%)
ARPKD	3 (4.2%)
Interstitial Nephritis	3 (4.2%)
Unknown	3 (4.2%)
IgA Nephropathy	1 (1.4%)
SLE	1(1.4%)
RPGN	1(1.4%)
**Induction**	
ATG	21 (29.2%)
Basiliximab	51 (70.8%)
**Maintenance immunosuppressants**	
Tac + MMF + Prednisone	70 (97.2%)
Tac + Azathoprime+Prednsione	1 (1.4%)
Tac + MMF	1 (1.4%)
**Positive Serology Test Results?**	
Yes, positive anti-SARS-CoV-2 spike protein IgG (Diasorin) + positive anti-SARS-CoV-2 N protein IgG (Abbot)	8(11.1%)
No, positive anti-SARS-CoV-2 spike protein IgG (Diasorin) + negative SARS-CoV-2 N protein IgG (Abbot)	1(1.4%)
No, negative for both tests	63(87.5%)
**Total population**	72(100%)

**Table 2:** Demographics and Characteristics of SARS-C0V-2 Serology-positive Patients

Cases	TST(m)	IS Regiment	Tac level (μ/L)	MMF/M2/d (mg)	Symptoms
Case 1	45	Tac/MMF/Pred	5.2	625	Asymptomatic
Case 2	32	Tac/MMF/Pred	5.4	620	Fever, cough, headache, SOT, diarrhea
Case 3	53.3	Tac/MMF/Pred	5.7	937.5	Fever, headache, cough, runny nose, anosmia and augesia
Case 4	15	Tac/MMF/Pred	7.7	648	Fever, diarrhea
Case 5	46	Tac/MMF/Pred	4.4	595	Asymptomatic
Case 6	40	Tac/MMF/Pred	4.8	769	cough
Case 7	45.9	Tac/MMF/Pred	4.8		Cough, runny nose, diarrhea
Case 8	45.6	Tac/AZA/Pred	6.9	652	Asymptomatic
Case 9	3.3	Tac/MMF/Pred	5.4	694	Asymptomatic

*TST* Time since transplant, *m* Months, *Tac* Tacrolimus, *MMF* Mycophenolate Mofetil, *M2* Surface area, *d* Day, *Pred* Prednisone, *AZA* Azathioprine, *IS* Immunosuppressant

The third patient presented to the ER with a history of fever (38.5 °C), cough, shortness of breath, myalgia and headache. PCR taken at that time was positive for COVID-19 (case 2). The fourth patient (case 3) had symptoms and had positive PCR results that were not disclosed before serology testing.

The median age of patients with detectable COVID-19 antibodies was 9 years (IQR, 6–13 years). A total of 77.8% (7 out 9) were male (Table 2). Seven patients were on triple maintenance immune suppressants consisting of tacrolimus, mycophenolate mofetil (MMF) and prednisone every other day. One patient was on azathioprine instead of MMF, and one patient was not on prednisone. The median time from transplant to testing positive was 3.8 years (IQR, 1.5–3.8). All but one patient had a household contact with confirmed COVID-19. Upon asking the eight asymptomatic children or their parents at the time of serology testing about history of COVID-19 symptoms in the last 3 months, 75% (6 of 8) of them reported mild symptoms. A total of 62.5% (5 of 8) reported history of fever, 50% (4 of 8) had history of cough, 2 had history of diarrhea (25%), and headache and loss of taste and smell were reported in one patient (12.5%) (Table 2).

### SARS-CoV-2 antibody test results

A total of 85 serological tests using both Diasorin **Liasion®** and Abbott were performed on 72 PKT recipients. We reported agreement between Abbott and Diasorin in 8 patients (88.9%). All patients with a positive PCR test except one had serological evidence of seroconversion (3/4). The patient who did not exhibit seroconversion received rituximab 4 months before her positive PCR result. She was first tested 70 days after her first positive PCR test and again at 90 days, at which point she was serologically negative. Notably, this patient received 4 doses of rituximab 4 months before her positive SARS-CoV-2 PCR, and her CD19 cell count at that time was 0 cell/μL. In one patient (case 9), the Abbott test was negative, but the Diasorin assay was positive. To ensure that this was not a false positive result, we repeated both tests after 1 week, and the results were the same. This patient’s PCR was negative, the patient was completely asymptomatic, and the parents denied any history of close contact with a COVID-19 patient.

From previous reports, we learned that most COVID-19 patients are IgG antibody positive by three weeks [12] and that antibodies start to decline after two to three months [13]. However, this is not the case with our young patients. Surprisingly, we had two patients whose IgG titers were above the test upper limit of detection (400 AU/ml). One patient had his test repeated 70 days after his mild symptoms (case 6), and the other had his test repeated 88 days after his symptoms (case 8). Overall, all patients had increasing titers over time (Table 3). The lowest titer detected was 32 AU/ml (Case 9). All patients with positive serological tests had their test repeat at least once except for two patients (cases 5 and 7). There was a clear increase in the serology titer over time, and all of them had positive antibodies until the end of the study. The median time from onset of symptoms or from PCR in asymptomatic patients to the first serological test was 31 days (IQR 19.5–49.5 days), and the median time to the last serological test was 75 days (IQR, 44.5–86.5 days).

**Table 3:** Kinetics of SARS-COV-2 IgG antibody-positive PKD patients using Diasorine Liasion® serological assays

Cases	PCR Status	Duration of +ve PCR(d)	Serology Test 1 (Au/ml)	Duration 1(d) post PCR/Symptoms	Serology Test 2 (Au/ml)	Duration 2(d)	Serology (3/4) (Au/ml)	Duration 3/4(d)	Households contact serology (Au/ml)
**1**	Positive	24	153	30	238	53	104	85	186
**2**	Positive	37	negative	7	negative	31	116/192	69/120	135
**3**	Positive	75	negative	44	31.1	75	N/D	N/D	20.4
**4**	Negative	–	185	27	135	83	N/D	N/D	25
**5**	N/D	–	223	57	N/D	N/D	N/D	N/D	38
**6**	Negative	–	216	12	297	33	> 400	70	38.3
**7**	N/D	–	98	32	N/D	N/D	N/D	N/D	N/D
**8**	Negative	–	234	55	311	67	> 400	88	38.3
**9**	Negative	–	41	U/K	32	N/D	N/D	N/D	N/D

*PCR* Polymerase Chain Reaction, *d* days, *N/D* Not Done, *U/K* unknown

We compared the antibody titer of our patients to the antibody titer of their immunocompetent parents or adult household contacts who requested to have their antibody titers checked using the same quantitative assay (Table 3). Interestingly, their results were much lower than those of their children except in case 1.

## Discussion

Most SARS-CoV-2 infections in children with intact immune responses are mild. However, severe infection can rarely occur [14]. The difference in disease severity between adult and pediatric populations might be related to a more robust innate immune response and higher levels of interleukin-17 and interferon gamma in children [15],, which play an important role in early disease containment. This difference in disease severity could also be explained by the fact that children are more prone to recurrent viral infection that can cause epigenetic changes in their trained immune system, making it more efficient at fighting the virus [16]. Immunocompromised patients are always at a higher risk of contracting severe viral and bacterial infections. Understanding the host immune response of the kidney transplant population to SARS-CoV-2 infection is evolving but remains unclear.

Herein, we reported our experience in using serological testing as a screening tool for COVID-19 PKT recipients. Of the 72 PKT recipients who underwent serological testing, the prevalence of COVID-19 based on two positive serological tests for SARS-COV-2 IgG was 11.1% (8 of 72). The patient with positive PCR and negative seroconversion was found to have profound B cell lymphopenia a few months before her infection, which could explain the lack of antibody production in this case. Few case reports from multiple sclerosis patients who were on B lymphocyte-depleting agents showed either negative SARS-CoV-2 seroconversion or an attenuated antibody response [17, 18]. However, no one knows how these patients would respond to vaccination. Another explanation for the negative seroconversion in this patient could be that she lost her antibodies to SARS-CoV-2 by the time she was tested.

It is well known that the early adaptive humoral immune response involving the development of neutralizing antiviral antibodies is an important mechanism in eliminating viral replication and providing protection from reinfection [19]. Our patients demonstrated high antibody titers against spike proteins tested by Diasorin assay, which was recently found to correlate well with neutralizing antibodies [20]. These findings may indicate that pediatric kidney transplant patients are capable of producing protective immunity post infection. In our series, all patients with positive serology, including the one with positive PCR results without seroconversion, were either asymptomatic or had mild symptoms not requiring hospital admission. None of them developed pneumonia or required oxygen supplementation. Our findings are consistent with what has been reported so far. A study done by Marlais et al., on 113 COVID-19 immunocompromised children with kidney disease, including 53 transplant patients, found that most of the children with kidney disease taking immunosuppressive medications have mild SARS-CoV-2 infection [21]. Another large multinational observational study performed by the Italian Society of Pediatric Nephrology included 1572 children with CKD or on immunosuppressants, of whom 288 (18%) were kidney transplant patients, none of them contracted severe SARS-CoV-2 infection during the study period [22].

Furthermore, a large multicenter seroprevalence study including 992 children of health care workers living in the UK found that 68 (6.8%) were positive for SARS-CoV-2 antibodies, 50% of them were asymptomatic, and the rest had only mild symptoms [23]. In another study that enrolled 13 patients with inflammatory bowel disease on immune suppressants who were diagnosed with COVID-19 based on a positive PCR, seroconversion occurred in 4 out of the 6 tested patients (66.7%), one patient had an equivocal serological test, and one did not exhibit seroconversion. Notably, 46.2% of the patients were completely asymptomatic despite no alteration in their baseline immunosuppressants; 53.8% were mildly symptomatic, and fever was the most common symptom [24].

After reviewing the limited available literature about SARS-COV-2 infection in the pediatric transplant population, we found that transplantation and an immunocompromised state do not increase the risk of ICU admission or mortality in the pediatric population. However, data in the adult transplant population showed contradictory results. In the pediatric transplant population, there is some evidence from multiple case series showing that immunosuppressant medications might have a beneficial effect. Bush et al. reported a case of a pediatric kidney transplant recipient who had a mild course of the disease despite being on immunosuppressant medications. In a series of 200 pediatric liver transplant patients in a large transplant center in Italy, 3 cases of COVID-19 were reported, and none of these patients had severe pneumonia or a severe course [25]. This observation led the author to conclude that immunosuppressant medications might have a protective role.

In a systematic review of 16 articles including children and adults with cancer and kidney, heart, liver and immunodeficiency patients, immunocompromised patients had similar and sometimes more favorable outcomes than the general population. However, this favorable outcome was not observed in cancer patients [26].

In our study, serological testing identified five patients with COVID-19 who otherwise would have been missed because of mild or asymptomatic disease. Although none of those five patients had severe symptoms or complications that required hospital attention, identifying them helped us better understand the full clinical spectrum of the disease in this vulnerable group. We learned that asymptomatic COVID-19 is not uncommon in the PKT population.

Out of the four patients with documented positive PCR, 75% seroconverted with relatively high antibody titers. Two patients had readings above the detectable upper limits of the test (> 400 AU/ml). Although these levels are very high, no one truly knew how these levels translate into protection from the virus. These findings were unexpected, as we already know that compared to the regular population, transplant patients who developed infection have a blunted immune response and a low rate of seroconversion [27].. The fact that our transplant patients mounted a strong humoral immune response to natural infection makes us believe that they will elicit the same, if not stronger, immune response with future vaccination when it becomes available for this age group. More interestingly, all patients who mounted rigorous immune responses to COVID-19 were still positive at a median time of 75 days (IQR, 69–83 days). One patient (case 2) was still positive for SARS-CoV-2 IgG at 120 days. The fact that all our positive serology patients had a history of contact with SARS-CoV-2 PCR-positive household members validates the positive serology results in our cohort. It also supports the hypothesis of possible household transmission among children that was addressed by Buonsenos et al [28]. The one patient (case 9) who had only one positive test was consider as negative. Of note, the parents’ antibody titers from approximately the same time as those assessed their children were significantly lower than those in their children except in one patient. These findings are in contrast to what has been reported by Pierce et al., who showed that serum neutralizing SARS-CoV-2 IgG antibodies were higher in adults than in children [15].

Importantly, no major modifications were made to the immunosuppressant medication in any PCR-positive patients except for one patient (case 3) in whom the MMF dose was reduced by half for one week. All patients but one developed a humoral immune response to COVID-19. This high rate of seroconversion in the PKT population has been replicated in adults and a few pediatric studies. In a recently published work by Prendecki et al., they found a relatively high seroprevalence rate of 10.4% in a cohort of adult kidney transplant recipients [29] In another small cohort of the PKT population that included 31 patients, seroconversion was reported in only one patient who was completely asymptomatic, and 3 patients had intermediate positive results [30]. Two adult kidney transplant patients reported seroconversion between days 19 and 23 after the reduction of immunosuppressants [31].. There was also documented seroconversion in another two adult kidney transplant patients who developed SARS-CoV-2 antibodies at 30 days post infection [32]. Moreover, a study performed by Hartzell et al. reported 100% seroconversion in 16 SARS-CoV-2-positive adult kidney transplant patients [33]. Furthermore, Choi et al. also reported a 100% seroconversion rate in 5 adult KTR patients [34]. Some of the current adult transplant guidelines including the guidelines from the Canadian society of transplantation pediatric group [35] suggest reduction or complete withdrawal of immunosuppressants. However, this suggestion was challenged by multiple studies that showed the ability of CNI to inhibit in vitro viral replication of SARS-CoV-2 infection independent of its immunosuppressant effects [36]. Furthermore, cyclosporine was successfully used to treat cases of secondary hemophagocytic lymphohistocytosis (HLH) that overlaps with the cytokine release storm, which seems to be the leading cause of mortality in COVID-19 patients [37].

To the best of our knowledge, our study is the first to report a relatively high seroprevalence of COVID-19 in the PKT population. This is the first study to test the humoral immune response to SARS-CoV-2 infection using a quantitative serological assay that correlates with neutralizing antibodies in this population. However, similar to all pediatric kidney transplant studies, we were limited by a small sample size, especially in this highly protected group. Moreover, the results cannot be generalized to all PKT patients, as the majority of the patients enrolled were more than one year post transplantation and were on low-dose maintenance immunosuppressants. The immune assays used were IgG based, so acute infection could have been missed. Importantly, all samples obtained were from Saudi children who might have different immunological backgrounds that can affect the immunoassay performance characteristics. For all these mentioned reasons, further larger studies with wider geographical samples and longer durations of antibody titer follow-up are needed.

## Conclusion

The seroprevalence of SARS-CoV-2 infection in our study is the highest reported among the PKT population so far. Screening PKT patients using serological testing was effective in identifying the whole spectrum of the disease, including asymptomatic patients. On the other hand, symptom-based screening for SARS-CoV-2 infection might not be an effective tool in identifying who should be tested, as asymptomatic infection is not uncommon and can lead to disease spread in hospital settings and in public. Most importantly, the humoral immune response to SARS-CoV-2 infection was rigorous and persistent in the PKT population, which will have positive implications for future vaccination. Immunosuppressants seem to not exert an additional risk for a more severe course in this population, and a reduction in immunosuppressants might not be necessary.

## Data Availability

The datasets used and/or analyzed during the current study are available from the corresponding author upon reasonable request.
